# Analysis of Two Complementary Single-Gene Deletion Mutant Libraries of *Salmonella* Typhimurium in Intraperitoneal Infection of BALB/c Mice

**DOI:** 10.3389/fmicb.2015.01455

**Published:** 2016-01-05

**Authors:** Cecilia A. Silva-Valenzuela, Roberto C. Molina-Quiroz, Prerak Desai, Camila Valenzuela, Steffen Porwollik, Ming Zhao, Robert M. Hoffman, Helene Andrews-Polymenis, Inés Contreras, Carlos A. Santiviago, Michael McClelland

**Affiliations:** ^1^Department of Microbiology and Molecular Genetics, University of California, IrvineIrvine, CA, USA; ^2^Departamento de Bioquímica y Biología Molecular, Facultad de Ciencias Químicas y Farmacéuticas, Universidad de ChileSantiago, Chile; ^3^Center for Adaptation Genetics and Drug Resistance, Tufts UniversityBoston, MA, USA; ^4^Anticancer Inc.San Diego, CA, USA; ^5^Department of Surgery, UC San Diego School of Medicine, University of California, San DiegoSan Diego, CA, USA; ^6^Department of Microbial Pathogenesis and Immunology, Texas A&M UniversityCollege Station, TX, USA

**Keywords:** *Salmonella*, deletion mutants, single-gene deletion library, IP infection, systemic colonization, BALB/c mice

## Abstract

Two pools of individual single gene deletion (SGD) mutants of *S*. Typhimurium 14028s encompassing deletions of 3,923 annotated non-essential ORFs and sRNAs were screened by intraperitoneal (IP) injection in BALB/c mice followed by recovery from spleen and liver 2 days post infection. The relative abundance of each mutant was measured by microarray hybridization. The two mutant libraries differed in the orientation of the antibiotic resistance cassettes (either sense-oriented Kan^R^, SGD-K, or antisense-oriented Cam^R^, SGD-C). Consistent systemic colonization defects were observed in both libraries and both organs for hundreds of mutants of genes previously reported to be important after IP injection in this animal model, and for about 100 new candidate genes required for systemic colonization. Four mutants with a range of apparent fitness defects were confirmed using competitive infections with the wild-type parental strain: Δ*STM0286*, Δ*STM0551*, Δ*STM2363*, and Δ*STM3356*. Two mutants, Δ*STM0286* and Δ*STM2363*, were then complemented *in trans* with a plasmid encoding an intact copy of the corresponding wild-type gene, and regained the ability to fully colonize BALB/c mice systemically. These results suggest the presence of many more undiscovered *Salmonella* genes with phenotypes in IP infection of BALB/c mice, and validate the libraries for application to other systems.

## Introduction

Salmonellosis is a food-borne disease caused by several serovars belonging to the genus *Salmonella.* Among them, *Salmonella enterica* ssp. *enterica* serotype Typhimurium (*S*. Typhimurium) is a broad host-range pathogen that causes gastroenteritis in humans and many other mammals and birds. In mice that are susceptible to systemic infection, such as BALB/c mice, *S*. Typhimurium causes a typhoid-like disease resembling infection of *S*. Typhi in humans (reviewed in Haraga et al., 2008).

*S*. Typhimurium infection begins with the ingestion of contaminated food or water. *Salmonella* reaches the ileum and invades epithelial host cells, inducing its own internalization (Galán, 1996, 1999). This process is triggered by effectors of the Type III Secretion System (T3SS) encoded in the *Salmonella* Pathogenicity Island 1 (SPI-1) (Galán, 1996, 1999; Lara-Tejero and Galán, 2009). In animals susceptible to systemic infection, *Salmonella* disseminates to deep organs such as liver and spleen. Effectors of the T3SS encoded in SPI-2 mediate intracellular growth by altering vesicular trafficking, allowing the establishment of the “*Salmonella*-containing vacuole” where bacteria actively replicate (reviewed in Hensel, 2004; Haraga et al., 2008; Lara-Tejero and Galán, 2009).

Several studies have been conducted to identify genes involved in *S*. Typhimurium pathogenesis using *in vitro* and *in vivo* models of infection. A number of these studies have used high-throughput analyses and signature-tagged mutagenesis where mutants are generated by random insertion of a transposon (Chan et al., 2003; Lawley et al., 2006; Chaudhuri et al., 2009). One of the biggest disadvantages of the use of random transposon libraries is that a large number of mutants have to be used in order to obtain a sufficient coverage for the genome of the bacterial species of interest. Random loss of selected mutants is a well-known complication of such approaches (Chan et al., 2003; Lawley et al., 2006; Chaudhuri et al., 2009; Silva et al., 2012). An alternative approach is to study mutants generated by the targeted deletion of genes of interest. However, due to the great effort to construct such mutants only a limited number of large libraries have been constructed. Such mutant collections have so far been established in *Escherichia coli* K12 (Baba et al., 2006; Yamamoto et al., 2009), *Acinetobacter baylyi* ADP1 (de Berardinis et al., 2008) and *S*. Typhimurium 14028 (Santiviago et al., 2009; Porwollik et al., 2014).

We performed a genetic screen of two single-gene-deletion (SGD) mutant libraries of *S.* Typhimurium. One library includes 3,690 mutants detectable on arrays, in which genes were replaced by a kanamycin-resistance cassette in the sense orientation (SGD-K). The other library was chloramphenicol resistant and contained 3,487 mutants (SGD-C) with the cassette placed in the antisense orientation. In total, deletion mutants of 3,923 genes were subjected to the screen of systemic infection of BALB/c, representing ~90% of 4,203 annotated non-essential *Salmonella* genes (Porwollik et al., 2014). In this work, we validate these collections for study of genes needed during infection, and identify many novel genes under selection in BALB/c mice after intraperitoneal (IP) infection. The two SGD libraries allowed the identification of hundreds of mutants previously known to have reduced competitiveness in organ colonization after IP injection. Novel candidate mutants involved in this process were also identified.

We observed that some mutants had a phenotype either only in the Kan or Cam library, as would happen with polarity effects on neighboring genes. Thus, the most confident candidates are the mutants supported by both libraries, without regard to cassette orientation. These complementary libraries will be of great utility to identify novel genes involved in less well-studied environments and conditions in the future.

## Results

### Identification of genes of *S.* Typhimurium involved in systemic colonization of BALB/c mice

**Figure 1:**
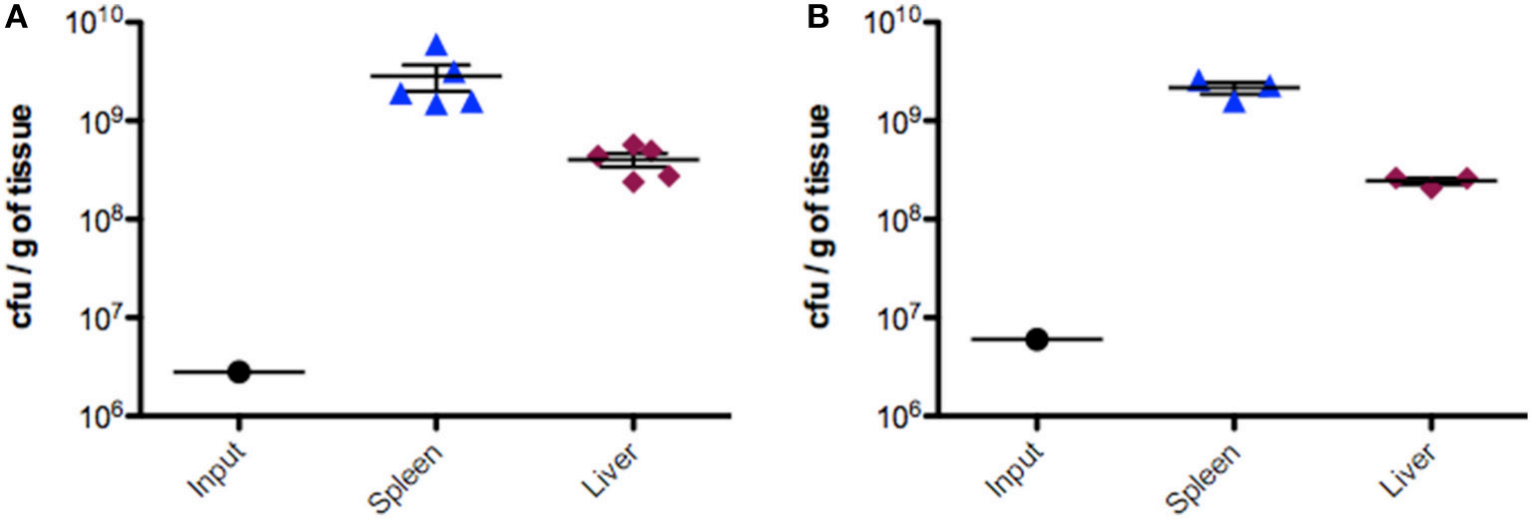
**Colonization of mice by IP delivery of SGD-K or SGD-C mutant libraries**. Groups of BALB/c mice (6–10 week old female) were infected by the IP route with ~6 × 10^6^ CFU of the SGD-K (**A**, left panel) or the SGD-C (**B**, right panel) mutant library in 200 μl PBS. Mice were sacrificed 2 days post infection; spleen and liver were removed, weighed, homogenized, and CFU counts were evaluated. Input values are the average number of bacteria inoculated into each animal.

Groups of BALB/c mice were injected intraperitoneally with ~6 × 10^6^ CFU of the pooled SGD-K or SGD-C mutant libraries (Porwollik et al., 2014). Bacteria were recovered from the spleens and livers 2 days post inoculation, sites where the bacterial population expands 100–1000 fold relative to the inoculum (Figure 1).

Mutants with altered fitness during infection were identified using a high-density Nimblegen tiling array for *Salmonella* (~387,000 oligos). Data for all 3,923 mutants detected on the arrays from the SGD-K and SGD-C input libraries are listed in Table S1. Their log_2_ fold change ratio (*M*-value) in both spleen and liver were converted to a rank order to permit comparison across different mice that might differ in the extent of replication of bacteria and, thus, the strength of selection. In Table S1, the *M* and FDR values of the top 500 candidates under negative selection are marked in red, while the top 250 under positive selection are marked in blue for each of the four tissue/library combinations. The FDRs of negatively selected genes are converted to negative numbers for convenience when sorting this table.

Taking into account that some of the mutants may be under selection in rich media, we screened both libraries for selection after five passages through Luria Bertani (LB) broth. All mutants under selection in this condition were identified. The selection was generally very mild in LB. The 160 top-ranked under the strongest negative selection and the 160 mutants under the strongest positive selection were annotated (Table S1).

Mutants were filtered for *M* < −1, rank order < 1000, and FDR < 0.2 in two or more conditions. Table S2 presents 224 candidate negatively selected mutants that survived this filter. The same filtering, but for positively selected mutants, was performed for *M* > 1, rank order in the top 1000, and FDR < 0.2. Table S3 presents these 63 candidates. The thresholds used are arbitrary and are chosen to identify some of the best and most consistently selected candidates. Some examples of mutants in genes known to be involved in infection after IP delivery and observed to be under apparent negative selection in both libraries and in both tissues in our screens are listed in Table 1. These included the *S*. Typhimurium-specific genes (compared to *E. coli*) required for *Salmonella* pathogenicity, such as the T3SS in SPI-2 (Shea et al., 1996; Cirillo et al., 1998; Chan et al., 2003; Lawley et al., 2006; Chaudhuri et al., 2009; Santiviago et al., 2009; Silva et al., 2012). Mutants in the T3SS encoded in SPI-1, its transcriptional regulators and its secreted effectors were also negatively selected, as previously observed (Chan et al., 2003; Lawley et al., 2006; Chaudhuri et al., 2009; Santiviago et al., 2009; Silva et al., 2012). Outside the five major *Salmonella* pathogenicity islands, mutants in SPI-6 appear to be under negative selection, as reported for gastrointestinal colonization and systemic spread of *S*. Typhimurium in mice (Mulder et al., 2012) and chickens (Pezoa et al., 2013). In agreement with previous reports, we also observed that mutants in genes *STM3119*-*STM3121* within SPI-13 appeared to be under negative selection in our IP screen (Haneda et al., 2009; Santiviago et al., 2009; Silva et al., 2012).

**Table 1:** **Examples of *Salmonella*-specific genes needed for systemic colonization in BALB/c mice**.

**Category**	**Number of mutants negatively selected**	**Genes**
SPI-1	16	*sitD, hilC, orgB, prgJ, prgI, sipB, sicA, spaR, spaQ, spaP, spaO, invI, invB, invA, invG, invF*
SPI-2	28	*ssrB, ssaB, ssaC, ssaD, ssaE, sseA, sseB, sscA, sseC, sseD, sseE, sseF, ssaG, STM1407, ssaI, STM1407, STM1410, ssaK, ssaL, ssaM, ssaV, ssaN, ssaO, ssaP, ssaQ, ssaR, ssaT, ssaU*
SPI-3	1	*mgtB*
SPI-6	4	*STM0278, STM0280, STM0286, STM0289*
SPI-13	3	*STM3119, STM3120, STM3121*
LPS	5	*rfbN, rfbI, rfbC, rfbD, rfbB*

Only genes linked to SPIs or LPS are shown. For a comprehensive list of top ranked genes needed for systemic colonization in BALB/c mice, see Table S2.

Mutants in other well-established pathogenicity-related genes that appeared to be consistently negatively selected in both libraries and both tissues in our study included genes related to the aromatic amino acid and purine biosynthesis pathways, exemplified by *aroE_2, aroA, aroK; purG, purF, purD, purM, purN, purE, purK, purA*, and *purC* (Edwards and Stocker, 1988; Chan et al., 2003; Betancor et al., 2005; Lawley et al., 2006; Chaudhuri et al., 2009; Santiviago et al., 2009; Silva et al., 2012); genes related to anaerobic metabolism such as the *nuo* (Turner et al., 2003; Chaudhuri et al., 2009; Silva et al., 2012) and *cyo* operons; the *tatC* gene encoding a component of the twin-arginine transport system (Reynolds et al., 2011; Silva et al., 2012); genes involved in lipopolysaccharide (LPS) biosynthesis such as *rfaK, rfaQ, rfaH, rfaB, rfaY, rfaL, rfaI, rfbD, rfbN, rfbC, rfbI, rfbB, rfbMUGFA*, and *wzxE* (Hoare et al., 2006; Silva et al., 2012); and genes related to the regulation of responses to external stimuli, such as *envZ, ompR, phoPQ*, and *rpoE* (Table S1; Gunn and Miller, 1996; Rakeman and Miller, 1999; Lawley et al., 2006; Chaudhuri et al., 2009; Silva et al., 2012). These findings validate our methodology.

### Confirmation of predicted phenotypes for selected mutants

Many additional mutants appeared to be under negative selection in BALB/c mice after IP inoculation. We chose four of these candidate mutants for further study, spread over a wide range in rank, including genes under apparently very mild negative selection (these genes are marked in yellow in Table S1). Three are deletions of genes specific to *Salmonella* (*STM0286, STM0551*, and *STM3356*) and one gene is shared with *E. coli* (*STM2363*) (Table 2). All are proximal to genes that when deleted also appeared to be under selection in our screen (Table S1). None of these mutants showed growth defects *in vitro* in LB, when compared to the wild-type strain (data for such genes is annotated in Table S1). According to their *M*-value and rank order, mutants in three of these genes (*STM0286, STM0551*, and *STM2363*) appeared to be under selection in both the SGD-K and SGD-C screens. The phenotype of the remaining mutant, Δ*STM3356*, was only present in the SGD-K screen, suggesting that this phenotype may result from a polar effect on a neighboring gene.

**Table 2:** **Initial selection phenotype data for mutants used for further studies**.

**Gene ID in *S*. Typhimurium LT2**	**SGD-K**				**SGD-C**				**Gene ID in *S*. Typhimurium 14028**	**Function (known or putative)**
	**Spleen**		**Liver**		**Spleen**		**Liver**			
	**M**	**FDR**	**M**	**FDR**	**M**	**FDR**	**M**	**FDR**		
*STM0286*	−0.6	−0.5	−1.4	−0.1	−2.7	0.0	0.0	1.0	*STM14_0334*	Putative cytoplasmic protein
*STM0551*	−0.8	−0.7	−1.9	−0.3	−3.9	0.0	−1.1	−0.1	*STM14_0643.RJ*	Putative diguanylate cyclase/phosphodiesterase domain
*STM2363*	−0.8	−0.2	−1.2	0.0	−4.7	0.0	−8.9	0.0	*STM14_2910*	Membrane protein required for colicin V production
*STM3356*	−1.4	−0.1	−1.8	0.0	1.5	0.1	0.9	0.6	*STM14_4048*	Putative cation transporter

The data are presented as M-value (log_2_ ratio of output to input) and FDR (false discovery rate). Red indicates that the mutant is under negative selection. Blue indicates that the mutant is under positive selection. Green indicates FDRs lower than 30%. M-value and rank order data for all assayed genes can be found in Table S1.

**Figure 2:**
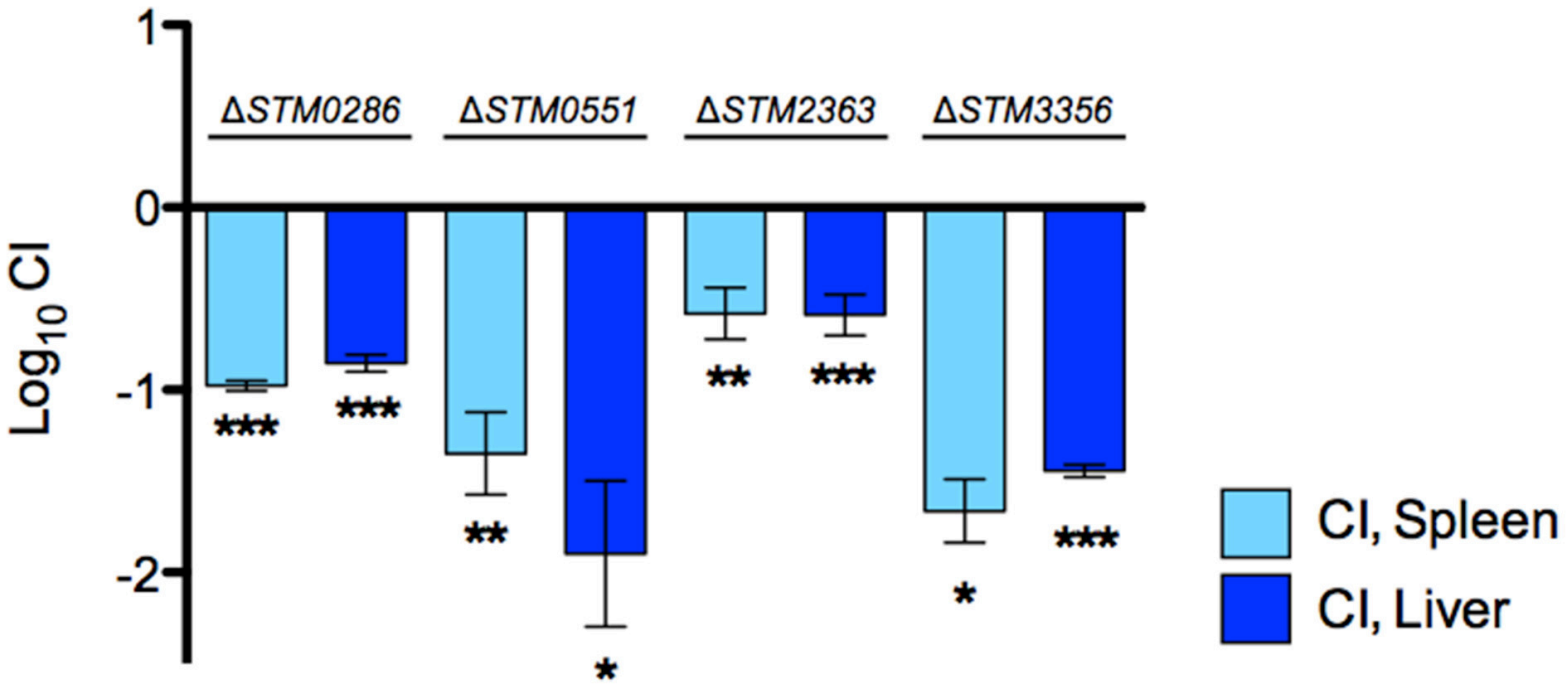
**Competition assays with individual novel candidates for *Salmonella* pathogenesis after IP delivery**. A 1:1 ratio of selected mutant strains and wild-type *S*. Typhimurium 14028s was inoculated by the IP route in groups of BALB/c mice and recovered from spleens and livers 2 days post infection. CI values were calculated as mean ratio of mutant versus wild type, normalized to the input ratios, and converted logarithmically. Error bars denote standard error. Statistical significance compared to the ideal value of 0 was determined using a two-tailed Student's *t*-test (^*^P < 0.05, ^**^P < 0.005, ^***^P < 0.001).

We re-tested each of these four SGD-K mutants using competitive infections with the wild-type strain in groups of BALB/c mice, to determine the competitive index (CI). These experiments confirmed the colonization defect of each of these mutants relative to the wild-type organism (Figure 2).

**Figure 3:**
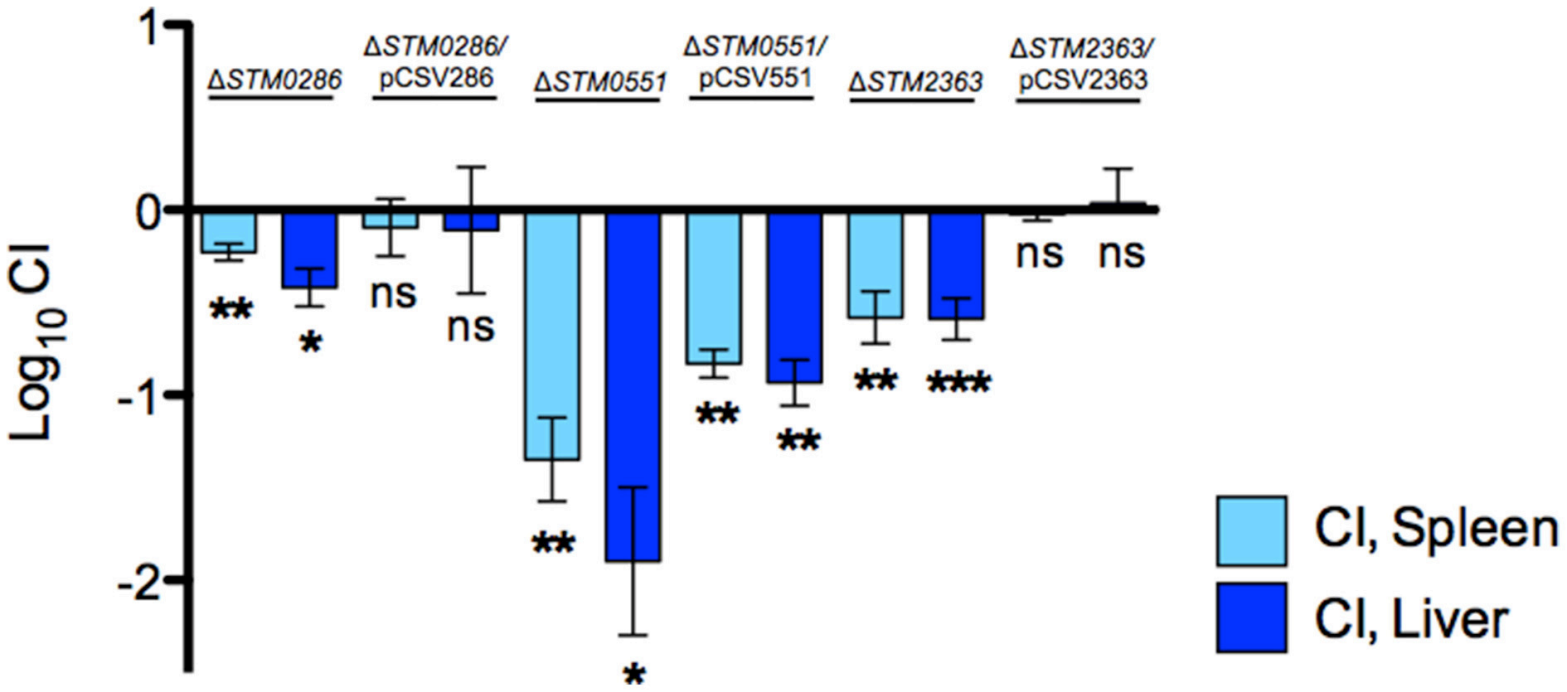
***STM0286* and *STM2363* are needed for colonization of BALB/c mice by *S*. Typhimurium after IP delivery**. A 1:1 ratio of selected mutants (or mutant strains complemented *in trans*) and wild-type *S*. Typhimurium 14028s was inoculated by the IP route in groups of BALB/c mice and recovered from spleens and livers 2 days post infection. Wild type and mutant strains harbor an empty copy of plasmid pBAD-TOPO as a control. CI values were calculated as mean ratio of mutant versus wild type, normalized to the input ratios, and converted logarithmically. Error bars denote standard error. Statistical significance compared to the ideal value of 0 was determined using a two-tailed Student's *t*-test (^*^P < 0.05, ^**^P < 0.005, ^***^P < 0.001, ns = not significant).

To directly link the defect in systemic infection to the selected genes, we performed complementation *in trans* of three mutants (Δ*STM0286*, Δ*STM0551*, and Δ*STM2363*), and tested these complemented strains in competitive infections. Mutant Δ*STM3356* was not further evaluated in complementation assays, as its phenotype was not consistent in the SGD-K and SGD-C screens. Complemented mutants Δ*STM0286* and Δ*STM2363* regained their ability to colonize systemic sites equally to the wild-type strain, whereas complementation of Δ*STM0551* only partially restored systemic colonization by the deletion mutant (Figure 3).

### Mutants Δ*STM0286*, Δ*STM0551*, and Δ*STM2363* do not have impaired internalization or survival in murine macrophages

**Figure 4:**
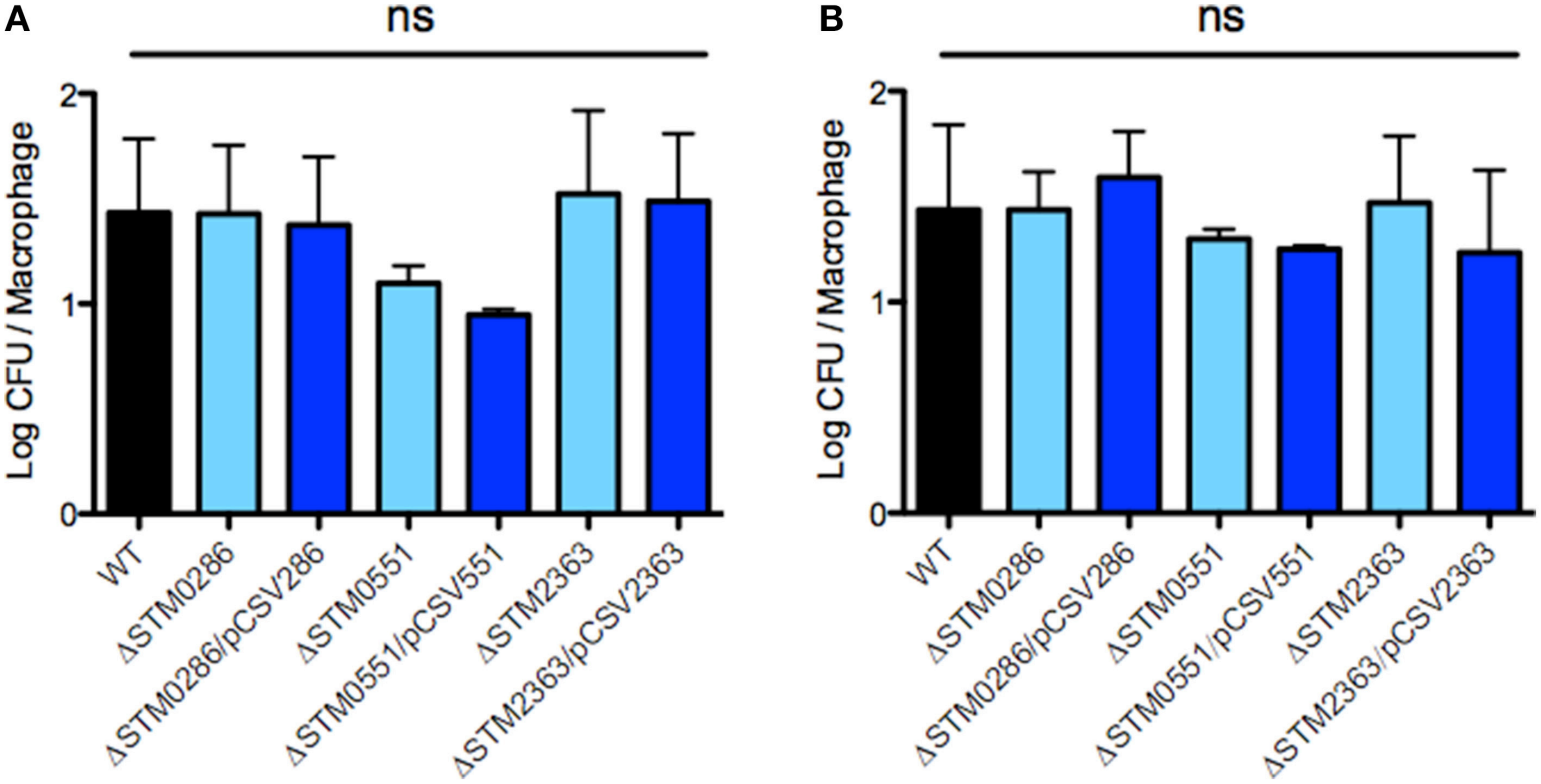
***STM0286*, *STM0551*, and *STM2363* are not needed for internalization or intracellular survival of *S*. Typhimurium within murine RAW264.7 macrophages**. Internalization at 1 h post infection (**A**, left panel) and survival at 20 h post infection (**B**, right panel) of wild-type *S*. Typhimurium 14028s and its isogenic mutants Δ*STM0286*, Δ*STM0551*, and Δ*STM2363* (and mutant strains complemented *in trans*) within RAW264.7 murine macrophages. Wild type and mutant strains harbor an empty copy of plasmid pBAD-TOPO as a control. Bars represent mean values, and error bars denote standard errors of at least three independent assays. Statistical significance was determined using a One-way ANOVA and the Bonferroni post-test. ns = not significant.

To determine if the colonization defects observed for the three mutants were due to a defect in intracellular survival within macrophages, we evaluated the wild-type strain, its derivative mutants Δ*STM0286*, Δ*STM0551*, or Δ*STM2363* and the same mutant strains complemented *in trans* for internalization and survival within RAW264.7 murine macrophages. Each of these mutants was internalized in macrophage and grew similarly to the wild type strain in our experiments (Figure 4). Thus, although mutants lacking *STM0286, STM0551*, or *STM2363* survive poorly during infection in mice, their survival defect may not be due to reduced internalization or replication inside macrophages, a primary niche for *Salmonella* growth in systemic sites.

## Discussion

In the past, the use of random transposon mutant libraries to identify genes under selection in an animal model by an array-based high-throughput screening method has been very helpful. However, when using such highly complex libraries, the dose of bacteria needed to encompass all the mutants must be large. An alternative, which allows a smaller dose of bacteria to be used, is to employ subsets of such a random library to analyze only a small fraction of the genes (Chaudhuri et al., 2009). An efficient means to screen mutants while keeping a low dose is the use of an ordered library of known transposon inserts (Cameron et al., 2008; Feinbaum et al., 2012; Held et al., 2012; Fey et al., 2013; Gallagher et al., 2013) or a library of targeted deletions (Santiviago et al., 2009; Elfenbein et al., 2013; Bogomolnaya et al., 2014; Porwollik et al., 2014), which allows reduction of the complexity of the pool without loss of genome coverage.

Here we employed a novel screening strategy using not one but two libraries of single-gene deletions. The mutants in our two libraries are constructed with either a Kan^R^ or a Cam^R^ resistance cassette and resistance gene promoters in opposite orientations. We speculate that when the corresponding mutants in both libraries give a similar result, the chance increases that the phenotype maps to that gene rather than to a genomic region nearby. Conversely, when there is a discrepancy in fitness for individual genes observed between the two libraries, a polar effect on nearby genes may be the reason. Thus, screening the two libraries provides additional clues about the selection process in each region of the genome.

The molecular mechanisms involved in the systemic colonization of mice by *S*. Typhimurium have been widely studied, yet our understanding of this process remains incomplete (Lawley et al., 2006; Chaudhuri et al., 2009; Santiviago et al., 2009). The libraries were subjected to IP infection of BALB/c mice. Arrays detected 3,690 and 3,487 mutants, respectively, in the Kan^R^ or Cam^R^ libraries, and a total of 3,923 mutants. We identified genes previously linked to *Salmonella* pathogenesis as well as new candidate genes needed for this process. Among the mutants experiencing the strongest apparent negative selection were many genes that have not been studied previously for a role in dissemination after IP delivery (Table S1, column U, indicating possible negative selection). We selected four of these genes (*STM0286, STM0551, STM2363*, and *STM3356*) with a range of severity in phenotype, for further study, including mild phenotypes similar to over 100 other new candidate mutants (Table 2). All four mutants had colonization defects as compared to the wild-type organism in confirmatory competitive infections, even the mutants that had relatively weak negative phenotypes in our pooled infection strategy (Figure 2). We have confirmed the new phenotypes for *STM0286* and *STM2363* (Figure 3).

*STM0286* (*sciT*) belongs to a Type VI Secretion System (T6SS) encoded in SPI-6 that is needed for *S*. Typhimurium virulence in mice after oral infection (Mulder et al., 2012), although its role during systemic infection has not been studied. ΔT6SS mutants of *S*. Typhimurium colonize poorly after IP and intragastric inoculation (Liu et al., 2012; Mulder et al., 2012; Pezoa et al., 2013).

*STM0551* is located between *fimY* and *fimW* and is not present in closely-related genera. This gene encodes a putative protein that includes a diguanylate-cyclase/phospodiesterase domain. These domains are well known to participate in the biosynthesis or degradation of cyclic dimeric GMP (c-di-GMP) (Simm et al., 2004), a bacterial second messenger important for cell-cycle regulation, differentiation, biofilm formation, motility and virulence (Simm et al., 2004; Tischler and Camilli, 2004; Römling et al., 2013). A mutant lacking *STM0551* was reported to be under negative selection in a genetic screen in BALB/c mice after IP infection, but this phenotype was not confirmed (Santiviago et al., 2009). In addition, *STM0551* is reportedly a repressor of type 1 fimbriae in *S*. Typhimurium, but the mechanism is still unclear (Wang et al., 2012). Although a mutant in this gene is under negative selection in our screen, complementation *in trans* only partially restored the systemic colonization defect, indicating either a polar effect of the mutation on adjacent genes, or failure of the complementing plasmid to produce an appropriate regulatory effect and/or amount of the protein.

Finally, *STM2363* (*cvpA*) is annotated as a putative membrane protein required for colicin V production and is also present in *E. coli, Citrobacter* and *Shigella*. Colicin V is an antibiotic small peptide (88 amino acids) secreted by an ABC transport system of some bacteria from the Enterobacteriaceae family to kill closely-related bacteria during niche/nutrient competition (Gilson et al., 1987, 1990; Zhang et al., 1995; Hwang et al., 1997; Gérard et al., 2005). If the annotation is accurate, a Δ*STM2363* mutant may be impaired in its ability to compete with the intestinal microbiota in the gastrointestinal phase of colonization. A separate functionality of *STM2363* could be indicated by the fact that a transposon insertion mutant in *cvpA* was impaired for biofilm and curli formation in uropathogenic *E. coli* (Hadjifrangiskou et al., 2012). This is the first report linking STM2363 to *Salmonella* pathogenesis.

Table S2 includes data for 224 mutants under negative selection. Several of these mutants are in genes already known to have a role in virulence (Table 1), but over 100 mutants are currently unreported as having negative phenotypes. Some of these apparent negative phenotypes are stronger than some of the mutants confirmed in this work. This indicates that many of these mutants may indeed be in genes that provide an advantage during infection.

Over 50 mutants showed a potentially *improved* ability to rapidly disseminate after IP delivery in our screen (Table S3). No mutants that improve short-term virulence after IP delivery are currently confirmed for *Salmonella*. This class of mutants could possibly involve genes that slow or modulate the infection process and are, for this reason, worthy of study in the future.

The present report demonstrates that the screening of the SGD-K and SGD-C libraries is a sensitive tool and allows the identification of new *Salmonella* pathogenesis genes even in a well-studied system, the mouse model. It will be of interest to investigate the role of the identified candidate genes in other animals of agricultural and epidemiological importance, such as chickens, cattle, and pigs, and in human *in vitro* systems, such as macrophages.

The many new potentially attenuated mutants that we have identified in this screen reveal new candidates for limiting factors in infection and could also serve to develop new strategies for vaccine development and for bacteria-mediated therapies to be performed in the future (Zhao et al., 2005; Arrach et al., 2010; Hoffman, 2012; Hoffman and Zhao, 2014). Among the other exciting future uses of these mutant resources will be the identification and confirmation of new gene phenotypes in less well-understood systems.

## Material and methods

### Bacterial strains, media, and growth conditions

*S*. Typhimurium wild-type strain 14028s was obtained from the ATCC. All strains and plasmids used in this study are listed in Table 3. Bacteria were routinely grown in Luria Bertani (LB) medium (10 g/l tryptone, 5 g/l yeast extract, 5 g/l NaCl) at 37°C with aeration. When required, LB medium was supplemented with ampicillin (Amp; 100 mg/l), tetracycline (Tet; 12.5 mg/l), streptomycin (Str; 100 mg/l), chloramphenicol (Cam; 20 mg/l), or kanamycin (Kan; 50 mg/l). Media were solidified by the addition of agar (15 g/l).

**Table 3:** **Strains used in the present study**.

**Strain**	**Genotype or relevant phenotype**	**Source**
***S**.* **Typhimurium**		
WT	*S*. Typhimurium 14028s wild type	Laboratory collection
14028s Δ*phoN*::Str	14028 Nal^R^ Δ*STM4319*::Str	Laboratory collection
14028s Δ*phoN*::Cam	14028 Nal^R^ Δ*STM4319*::Cam	Laboratory collection
14028s Δ*phoN*::Tet	14028 Nal^R^ Δ*STM4319*::Tet	Laboratory collection
Δ*STM0286*	Δ*STM0286*::Kan	SGD-K
Δ*STM0551*	Δ*STM0551*::Kan	SGD-K
Δ*STM2363*	Δ*STM2363*::Kan	SGD-K
Δ*STM3356*	Δ*STM3356*::Kan	SGD-K
Δ*STM0286*/pCSV286	Δ*STM0286*::Kan carrying pBAD-TOPO::*STM0286*	Present study
Δ*STM0551*/pCSV551	Δ*STM0551*::Kan carrying pBAD-TOPO::*STM0551*	Present study
Δ*STM2363*/pCSV2363	Δ*STM2363*::Kan carrying pBAD-TOPO::*STM2363*	Present study
***Escherichia coli***		
TOP10	K12 F^−^Φ80*lacZ*ΔM15 Δ(*lacZYA*-*argF*)U169 *deoR recA1* *endA1 hsdR17*($r_{k}^{-}$, $m_{k}^{+}$) *phoA* *supE44 thi-1 gyrA96 relA1* λ^−^	Life Technologies

### Standard DNA techniques

Total genomic DNA was obtained from overnight bacterial cultures using the GenElute Bacterial Genomic DNA Kit (Sigma-Aldrich) according to the manufacturer's instructions. Plasmid DNA was obtained from overnight cultures using the QIAprep Spin Miniprep Kit (QIAGEN), according to the manufacturer's instructions. DNA samples were routinely analyzed by electrophoresis in 1% agarose gels (prepared in 1X Tris-acetate-EDTA buffer) and visualized under UV light after ethidium bromide staining. Primers for PCR amplification were designed based on the reported sequence of *S*. Typhimurium 14028s (Jarvik et al., 2009) (Table S4). When required, PCR products were purified using the QIAquick PCR Purification Kit (QIAGEN) as recommended by the manufacturer, and eluted with nuclease-free water.

### Studies in BALB/c mice

All mouse studies were conducted at AntiCancer Inc. (San Diego, CA) with an Institutional Animal Care and Use Committee (IACUC)-protocol specifically approved for this study and in accordance with the principles and procedures outlined in the National Institute of Health Guide for the Care and Use of Animals under Assurance Number A3873-1.

A frozen glycerol aliquot of the pooled SGD-K or SGD-C mutant library was used to inoculate 25 ml of LB-Kan. Groups of 3-5 BALB/c mice (6–10 week-old female) were infected by the IP route with ~6 × 10^6^ CFU of each library in 200 μl of PBS. Samples of the inoculated material (*input library*) were kept frozen until further use. Mice were sacrificed 2 days post infection; the spleen and liver were removed and homogenized, and then CFU counts were determined. The remaining homogenate was used to inoculate 25 ml of LB-Kan or LB-Cam for overnight growth with agitation at 37°C (*output library*). The bacteria were pelleted, washed and stored frozen until further use.

Sample selection for further analyses was dependent on fluctuation tests to measure bottlenecks (Santiviago et al., 2009; Elfenbein et al., 2013). To do this, the SGD-C mutant pool for inoculations was spiked 1/20,000 with strain 14028s Δ*phoN*::Str. The SGD-K mutant pool for inoculations was spiked 1/10,000 with strain 14028s Δ*phoN*::Cam and 1/100,000 with strain 14028s Δ*phoN*::Tet. A fluctuation of up to 8 times was considered within biological range. A majority of samples complied with this criterion.

For phenotype confirmation of individual mutants, competition assays with wild-type *S*. Typhimurium 14028s in a 1:1 ratio inoculum were performed to determine the Competition Index (CI). CI values were calculated as a mean ratio of mutant versus wild type in the output, normalized to the input ratio: (mutant output/wild-type output)/(mutant input/wild-type input) and converted logarithmically as described in Silva et al. (2012). For complementation assays, the selected mutations were first transduced to the wild-type background using phage P22 HT105/1 *int*-201 before complementation *in trans*. Groups of 3–5 BALB/c mice (6–10 week-old female) were inoculated by the IP route with ~10^6^ CFU of the mixture in 100 μl PBS. Mice were euthanized 2 days post infection and CFU counts were obtained from spleens and livers. CI values were calculated as a mean ratio of mutant versus wild type, normalized to the input ratio and converted logarithmically. Statistical significance was determined using a two-tailed Student's *t*-test.

### Labeling of DNA adjacent to transposon insertions and microarray analysis

The DNA adjacent to the deletion in each mutant from the input and output libraries was specifically amplified as described (Santiviago et al., 2009), with modifications. Genomic DNA was prepared and subjected to shearing by sonication. Then, polyA tails were added to the DNA fragments using terminal transferase (TdT) following the instructions of the manufacturer (NEB). Finally, the product was purified using the QIAquick PCR purification kit (QIAGEN).

A nested PCR strategy was used to amplify only the polyA-tailed fragments containing the antibiotic-resistance cassette carrying the P_T7_ and the genomic DNA downstream of the insertion (Santiviago et al., 2009). In the first amplification reaction, 500 ng of purified polyA-tailed DNA were used as the template for a PCR reaction using primers FRT_Out3_1 and CCT24VN (Table S4). In the second amplification step, a nested PCR was performed using 1 μl of amplified product from the initial PCR as template in a total volume of 50 μl. Internal primer FRT_Out3_2 (Table S4) and primer CCT24VN were then used for PCR, as described (Santiviago et al., 2009).

An aliquot of the nested PCR reaction product was used as template for an *in vitro* transcription reaction using the AmpliScribe T7 transcription kit (Epicentre), following the manufacturer's protocol. During this process, Cy5-UTP or Cy3-UTP was added for the generation of labeled RNA. The remaining template DNA was digested with RNase-free DNase (Epicentre). The RNA was purified with the RNeasy Mini Kit (QIAGEN) and used for hybridization.

### Hybridization and data analysis

A total of 4 μg of labeled RNA was hybridized onto high-density Nimblegen tiling arrays (~387,000 oligos). The mixture of probes was combined with 39 μl of Nimblegen hybridization buffer (Roche Diagnostics) and then denatured at 95°C for 5 min. The labeled samples were hybridized to the *Salmonella* microarray at 42°C in a water bath, overnight. After incubation, slides were washed and scanned using the Axon GenePix 4000B laser scanner with Genepix Pro 6.0 software. Fluorescence signal intensities were quantified using NimbleScan 2.4 software (Roche Diagnostics).

Intensities for five probes following the 3-prime insertion site for each mutant were extracted using custom Perl scripts. Data for all samples were background-corrected and quantile normalized in Bioconductor. Normalized intensities for the five probes were summarized by calculating the median, which was used in further statistical analysis. Limma (Smyth, 2005) was used to calculate *M*-values and FDR associated with each mutant, between input and output samples. Then, mutants were sorted and numbered by rank order by *M*-value, from negative to positive.

### Cloning of genes for complementation assays

Primers STM0286_*BamHI*_out5, STM0286_*EcoRI*_out3, STM0551_*BamHI*_out5, STM 0551_*EcoRI*_out3, STM2363_out5_F_*BamHI*, STM2363_out3_R_*EcoRI* (Table S4) were used to amplify the selected genes of the *S*. Typhimurium strain 14028s genome. The PCR product was purified and ligated to expression vector pBAD TOPO TA (Life Technologies). Plasmids were transformed into chemically competent TOP10 *E. coli* according to the manufacturer's instructions. The presence of the insert in each plasmid was confirmed by PCR amplification. Then, each plasmid was transformed into its corresponding *S*. Typhimurium 14028s mutant.

### Cell-culture conditions and gentamicin protection assays using RAW264.7 macrophages

RAW264.7 murine macrophages were routinely maintained in DMEM (HyClone) medium supplemented with 10% inactivated fetal bovine serum (HyClone) (DMEM-FBS) at 37°C in a 5% CO_2_ atmosphere. Monolayers for infection were prepared the day prior to infection by seeding 10^5^ cells and incubating at 37°C for 20 h in 5% CO_2_. Bacteria were grown with aeration to an OD_600_ of 0.6–0.8, washed, suspended in 100 μl of DMEM-FBS, and added to confluent RAW264.7 macrophages at a multiplicity of infection of ~50 bacteria/cell. After 1 h of infection, macrophages were washed with PBS and incubated for 1 or 20 h in DMEM-FBS containing gentamicin (50 μg/ml), to kill extracellular bacteria. After incubation, cells were washed and lysed with 0.5% deoxycholate. Intracellular CFUs were determined by serial dilution and plating. Experiments were conducted at least in duplicate. The statistical significance of observed differences was determined using the One-way analysis of variance (ANOVA) and the Bonferroni post-test.

## Author contributions

Conceived and designed the experiments: MM, CASi, CASa, IC. Performed the experiments: CASi, RM, CV, PD. Analyzed the data: PD, CASi, CASa, MM. Contributed reagents/materials/analysis tools: MM, CASi, RH, PD, SP, IC, CASa, HA, MZ. Wrote the paper: MM, CASi, CASa, HA. RH and SP help revise the manuscript. All authors read and approved the final manuscript.

## Funding

This work was supported in part by FONDECYT grants 1110172 and 1140754; NIH grants AI039557, AI052237, AI073971, AI075093, AI077645 and AI083646; USDA 2009-03579-30127, 2011-67017-30127, BARD 2014-2016, CDMRP BCRP W81XWH-08-1-0720, the Center for Produce Safety, the American Meat Institute, and the University of California Cancer Research Coordinating Committee. CASi was supported by fellowships from CONICYT (21090260 and 24110001) and from the Sciences, Technology and Innovation Program for the Americas (MECESUP grant UCH0717). CV was supported by CONICYT fellowship 21140615.

### Conflict of interest statement

The authors declare that the research was conducted in the absence of any commercial or financial relationships that could be construed as a potential conflict of interest.
