# Supplementary material for: Analysis of Two Complementary Single-Gene Deletion Mutant Libraries of Salmonella Typhimurium in Intraperitoneal Infection of BALB/c Mice
Source: Front Microbiol. 2016 Jan 5;6:1455. doi: 10.3389/fmicb.2015.01455 (PMC4700939; doi:10.3389/fmicb.2015.01455)
Supplement: Supplementary file 4 [file Table4.pdf]

Table S4. Primers used in this study.

**Primer**	**Sequence**
**Amplification for hybridization**	
$CCT_{24}VN$	CCTTTTTTTTTTTTTTTTTTTTTTTTTVN
FRT-Out3_1	TTCCTATACTTTCTAGAGAA
FRT-Out3_2	TAGGAACTTCGGAATAGGAA
**Cloning of *STM0286, STM0551 and STM2363***	
STM0286_ *BamHI*_out5	GTCTGGATCCGCAGATCGCTTCGCTGCTGAC
STM0286_ *EcoRI*_out3	TTCTGAATTCTCCTGCACAGGCAGACATAAAC
STM0551_*BamHI*_out5	GTCTGGATCCTCACTTAACTTTTTACAAGG
STM0551_*EcoRI*_out3	TTCTGAATTCCCTGCTTTTTCCATTGCT
STM2363_*BamHI*_out5	GTCTGGATCCGAGATAAAAAGTGTGATGGC
STM2363 _*EcoRI*_out3	TTCTGAATTCTAAGCCACATCTCAGAACCTG
pBAD-F	ATGCCATAGCATTTTTATCCA
pBAD-R	GATTTAATCTGTATCAGG
